# Effect of Psychosexual Counseling Based on the Schover Bio‐Psycho‐Social Model on Sexual Self‐Concept and Sexual Distress in Women After Hysterectomy: A Randomized Controlled Trial

**DOI:** 10.1002/hsr2.72526

**Published:** 2026-06-22

**Authors:** Zeinab Maloum, Soheila Rabiepoor, Samira Barjasteh

**Affiliations:** ^1^ Student Research Committee, Urmia University of Medical Sciences Urmia Iran; ^2^ Reproductive Health Researcher Center, Clinical Research Center, Urmia University of Medical Sciences Urmia Iran

**Keywords:** biopsychosocial model, hysterectomy, psychosexual counseling, sexual distress, sexual self‐concept

## Abstract

**Background and Aims:**

Hysterectomy may lead to psychological and sexual challenges that extend beyond physical recovery. This randomized controlled trial evaluated the effect of psychosexual counseling based on the Schover bio‐psycho‐social model on sexual self‐concept (primary outcome) and sexual distress among women after hysterectomy.

**Methods:**

In this parallel randomized controlled trial, 100 married women aged 18–49 years who had undergone total or subtotal hysterectomy for benign conditions were randomly assigned (1:1) to an intervention group receiving four weekly 90‐min group counseling sessions based on the Schover model or to a control group receiving routine postoperative care. Sexual self‐concept (MSSQ‐SF) and sexual distress (FSDS‐R) were assessed at baseline and 1‐month post‐intervention. Analysis of covariance (ANCOVA) adjusted for baseline scores was performed (SPSS v22; *α* = 0.05).

**Results:**

After adjustment, significant between‐group differences were observed. The intervention group showed higher adjusted mean sexual self‐concept scores compared with controls (51.3 vs. 44.0), *F*_(1, 97)_ = 45.82, *p* < 0.001, η²ₚ = 0.32 (95% CI = [0.19, 0.46]). Sexual distress scores were significantly lower in the intervention group than in controls (29.1 vs. 42.0), *F*_(1, 97)_ = 53.21, *p* < 0.001, η²ₚ = 0.35 (95% CI = [0.22, 0.48]).

**Conclusion:**

Psychosexual counseling grounded in the Schover bio‐psycho‐social model significantly improved sexual self‐concept and reduced sexual distress among women after hysterectomy. Incorporating structured psychosexual counseling into postoperative care may support sexual rehabilitation in this population.

## 1 Introduction

Hysterectomy, the surgical removal of the uterus, is one of the most frequently performed gynecological operations worldwide and is categorized as partial, total, or radical hysterectomy [1]. It ranks as the second most common major surgical procedure among women, with nearly one in three undergoing it by the age of sixty and over 600,000 operations performed annually in the United States [2, 3]. In Iran, hysterectomy accounts for approximately 15%–20% of gynecological surgeries [4]. While the procedure effectively treats life‐threatening uterine disorders such as leiomyomas, abnormal uterine bleeding, endometriosis, uterine prolapse, cervical dysplasia, adenomyosis, and endometrial hyperplasia [5], it can also lead to complications depending on the surgical route—vaginal, abdominal, or laparoscopic—and the technique used. The most common complications include surgical site infection, venous thromboembolism, urinary and gastrointestinal tract injury, bleeding, nerve damage, vaginal cuff dehiscence, and vesicovaginal fistula [6].

Although hysterectomy provides substantial medical benefits, its long‐term effects on vaginal length, pelvic innervation, and sexual functioning remain controversial [7]. The uterus is not only a reproductive organ but also symbolically associated with femininity, sexual identity, and aesthetic body image in many cultures [8]. The loss of the uterus may therefore be perceived as a loss of womanhood, leading to disturbances in emotional stability and sexual confidence [9, 10]. Sexual complaints following hysterectomy often include decreased libido, reduced frequency of intercourse, impaired orgasmic capacity, diminished vaginal sensitivity, dyspareunia, feelings of rejection from the spouse, and decreased sexual satisfaction [11, 12, 13]. Anatomically, removal of the cervix and upper vagina can disrupt the uterovaginal nerve plexus, which contributes to lubrication and orgasmic response, thereby altering sexual arousal and pleasure [14, 15].

Beyond biological mechanisms, psychological and sociocultural factors play a critical role in shaping sexual experiences after hysterectomy [16]. Women may internalize negative beliefs about femininity, aging, and attractiveness, leading to low sexual self‐esteem and avoidance of intimacy [17]. Although hysterectomy effectively treats benign uterine disorders, its long‐term psychological and sexual consequences remain debated [18]. In conservative societies, especially across the Middle East, misconceptions linking hysterectomy with loss of womanhood exacerbate anxiety and marital strain, underscoring the need for culturally sensitive psychosexual interventions [19, 20].

Sexual self‐concept, defined as an individual's cognitive and emotional perception of herself as a sexual being, constitutes the foundation of sexual motivation and satisfaction and is a predictor of sexual behavior [21]. In contrast, sexual distress refers to a set of negative emotional reactions such as anxiety, frustration, and inadequacy regarding sexual performance, often resulting in impaired sexual relationships and reduced quality of life [22]. Shame and social taboos may prevent women from discussing their sexual problems openly, manifesting instead as anxiety, depressive symptoms, pelvic pain, dyspareunia, or vaginal dryness [23].

Despite the prevalence of these difficulties, most interventions for women after hysterectomy have concentrated on physical recovery, hormonal replacement, or surgical outcomes, neglecting the intertwined psychological and social factors that influence sexual well‐being [24]. This limited focus underscore the need for interventions that address both emotional and relational dimensions of sexual rehabilitation [25].

The bio‐psycho‐social model, introduced by Engel [26] and further operationalized in psychosexual therapy by Lisa M. Schover, provides a comprehensive framework for understanding and treating sexual dysfunctions through the integration of biological, psychological, and social components [27]. The Schover psychosexual counseling model, derived from this framework, emphasizes individualized assessment of sexual needs, exploration of cognitive and emotional barriers, correction of maladaptive beliefs, and promotion of effective partner communication. This model provides a structured, intervention‐oriented application of Engel's broader biopsychosocial framework in psychosexual counseling.

Given the scarcity of culturally adapted psychosexual counseling programs in Iran and the limited evidence on the effectiveness of bio‐psycho‐social interventions for women after hysterectomy, this study aimed to evaluate the impact of psychosexual counseling based on the Schover bio‐psycho‐social model on sexual self‐concept and sexual distress among women who underwent hysterectomy for benign conditions. It was hypothesized that the intervention would significantly enhance sexual self‐concept and reduce sexual distress compared with standard postoperative care.

## 2 Methods

### 2.1 Study Design and Setting

This randomized controlled clinical trial with a parallel design was conducted between June 2023 and February 2024 at Kowsar Women's Comprehensive Hospital, a tertiary referral center affiliated with Urmia University of Medical Sciences (Urmia, Iran). The study adhered to the CONSORT 2010 guidelines for randomized trials. Ethical approval was obtained from the Research Ethics Committee of Urmia University of Medical Sciences (IR.UMSU.REC.1402.025), and the trial was prospectively registered in the Iranian Registry of Clinical Trials (IRCT20230507058109N1). All procedures were performed in accordance with the Declaration of Helsinki. The primary outcome was sexual self‐concept, and the secondary outcome was sexual distress.

### 2.2 Participants and Eligibility Criteria

Participants were married women aged 18–49 years who had undergone total or subtotal hysterectomy for non‐malignant indications within the previous 3–6 months.

Inclusion criteria were: (a) living with their husband, (b) literacy in reading and writing, (c) no menopause prior to hysterectomy, (d) not breastfeeding, and (e) not having participated in similar psychological or sexual interventions within the last 6 months. Exclusion criteria included: (a) any diagnosed psychiatric or sexual disorder in either partner. Participants who missed more than one counseling session, withdrew consent, or relocated during the intervention period were considered lost to follow‐up and were handled according to the intention‐to‐treat principle.

Eligible participants were identified from surgical records and contacted by telephone to verify eligibility. After providing written informed consent, participants completed baseline questionnaires assessing demographic variables, sexual self‐concept, and sexual distress.

### 2.3 Sample Size Determination

Sample size was calculated using G*Power 3.1 based on data from Bolghan et al. (2021) [28], assuming *α* = 0.05, power = 0.80, and an effect size of 0.60 for the main outcome (sexual self‐concept). The required sample was 42 per group; accounting for an anticipated 20% attrition rate, 50 participants were included in each group (*N* = 100).

### 2.4 Randomization and Blinding

Participants were randomized (1:1) to the intervention or control group using a simple randomization method (even/odd sealed opaque envelopes). Randomization was performed by an independent researcher not involved in data collection or analysis. Given the behavioral nature of the intervention, blinding of participants and facilitators was not feasible; however, the data analyst was blinded to group assignments. The use of simple randomization was justified by the homogeneity of participants and the equal group sizes.

### 2.5 Intervention Protocol

The intervention followed the Schover bio‐psycho‐social counseling model [27], emphasizing the cognitive, emotional, behavioral, and relational dimensions of sexual health.

Each participant in the intervention group attended four 90‐min group counseling sessions, conducted weekly over 1 month (7–8 women per group).

Sessions were co‐facilitated by a faculty member with a PhD in Reproductive and Sexual Health, who has clinical and research experience in sexual health and has completed specialized training in psychosexual counseling, and a master's student in midwifery counseling trained under her supervision. The faculty member supervised the sessions to ensure adherence to the counseling protocol. Fidelity to the intervention protocol was monitored using a standardized checklist, and attendance exceeded 90%.

The session content was structured as follows:

- **Session 1:** Introducing hysterectomy and its physical and psychological consequences, establishing a safe space for discussion, and initiating dialogue about its impact on sexual relationships.
- **Session 2:** Identifying psychological factors such as anxiety, depression, and body image concerns, and examining their influence on sexual relationships and functioning.
- **Session 3:** Exploring physiological and medical factors, including hormonal changes, vaginal dryness, and dyspareunia, and discussing relevant medical and behavioral management strategies.
- **Session 4:** Addressing social and relational influences, including partner support, socioeconomic conditions, family dynamics, media, and cultural or religious beliefs, and their role in shaping post‐hysterectomy sexual relationships.

Educational materials, including PowerPoint presentations, printed pamphlets, and structured group discussions, were employed to encourage participant engagement and reinforce learning objectives. The control group received routine postoperative care according to hospital protocol, consisting of scheduled follow‐up visits for postoperative evaluation and management of patient‐reported symptoms, without any structured psychosexual counseling. Follow‐up assessments were conducted 1 month after the final session for both groups using the same evaluation instruments.

### 2.6 Data Collection Instruments

1. **Demographic Questionnaire**—Collected information on age, duration of marriage, number of pregnancies, education, occupation, socioeconomic status, type of surgery, and reason for hysterectomy.
2. **Multidimensional Sexual Self‐Concept Questionnaire (MSSQ–Short Form)**—Developed by Snell (1998) [29] and revised by Pantin et al. [30], comprising 20 items across four domains (sexual self‐efficacy, sexual anxiety, sexual satisfaction, and sexual desire), rated on a 5‐point Likert scale. The Persian version showed strong psychometric properties (CVI = 0.89, CVR = 0.82, Cronbach's *α* = 0.88) [31].
3. **Female Sexual Distress Scale–Revised (FSDS‐R)**—Developed by Derogatis et al. (2008) [32], containing 13 items rated from 0 (“*never*”) to 4 (“*always*”). Scores ≥ 11 indicate clinically significant sexual distress. The Persian version demonstrated high internal consistency (Cronbach's *α* = 0.91) [33].

### 2.7 Data Analysis

All analyses were conducted using SPSS version 22 (IBM Corp., Armonk, NY, USA). Normality of continuous variables was assessed using the Kolmogorov–Smirnov test. Data are presented as mean ± standard deviation (SD) for normally distributed variables and as frequency (percentage) for categorical variables. Between‐group comparisons at baseline and post‐intervention were performed using independent *t*‐tests or Mann–Whitney *U*‐tests, as appropriate. Analysis of covariance (ANCOVA) was used to evaluate intervention effects while adjusting for baseline values. Assumptions of normality, linearity, and homogeneity of variances were assessed prior to analysis. Effect sizes were reported as partial eta squared (η²) with 95% confidence intervals (CI). All analyses followed the intention‐to‐treat principle. All tests were two‐sided, and statistical significance was set at *α* = 0.05.

## 3 Results

### 3.1 Participant Characteristics

**Figure 1:**
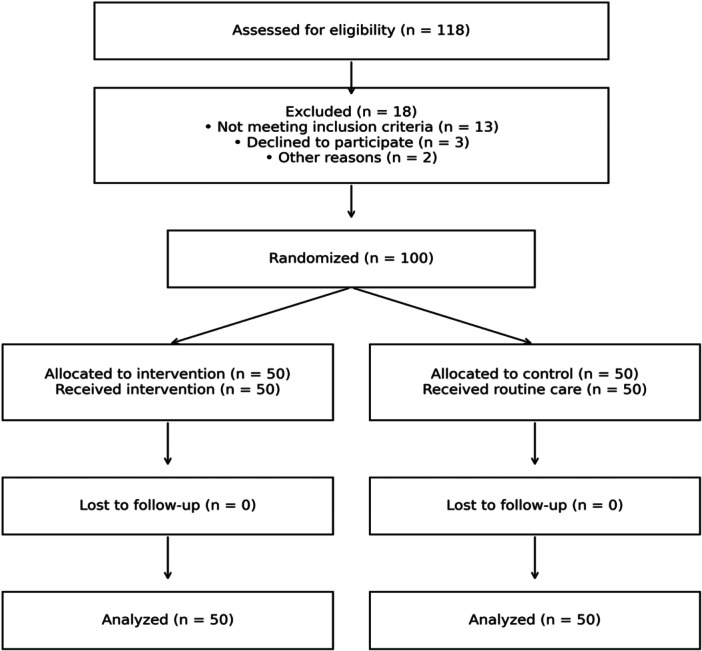
CONSORT flow diagram. CONSORT flow diagram illustrating participant recruitment, allocation, follow‐up, and analysis.

Of 118 screened candidates, 100 eligible women were randomized equally into intervention and control groups. The participant flow through the study is presented in Figure 1.

Baseline comparisons indicated that the two groups had broadly similar demographic and obstetric characteristics (Tables 1, 2, 3, 4), with no statistically significant differences detected between groups. The Kolmogorov–Smirnov test indicated that age at marriage and duration of marriage followed a normal distribution, while the other continuous variables were non‐normally distributed; therefore, independent *t*‐tests or Mann–Whitney *U*‐tests were applied as appropriate.

**Table 1:** Assessment of normality for continuous variables using the Kolmogorov–Smirnov test.

Variable	K–S statistic	*p* value	Distribution
Age	1.672	*p* = 007	Non‐normal
Age at marriage	1.277	*p* = 077	Normal
Duration of marriage	1.007	*p* = 263	Normal
Age of disease onset	1.750	*p* = 004	Non‐normal
Duration of illness	2.774	*p* < 0.001	Non‐normal
Number of pregnancies	2.613	*p* < 001	Non‐normal
Number of children	2.445	*p* < 001	Non‐normal
Number of births	2.446	*p* < 001	Non‐normal

*Note:* Normality confirmed for age at marriage and duration of marriage (*p* > 0.05); other variables were non‐normal (*p* < 0.05).

**Table 2:** Comparison of continuous demographic characteristics between intervention and control groups.

Variable	Group	Mean ± SD	*Z* value	*p* value
Age (years)	Intervention	42.44 ± 4.24	−0.721	*p* = 0.47
	Control	42.98 ± 4.26		
Age of disease onset (years)	Intervention	39.88 ± 5.06	−1.236	*p* = 0.22
	Control	41.36 ± 3.34		
Duration of illness (years)	Intervention	2.76 ± 1.92	−0.574	*p* = 0.57
	Control	2.44 ± 1.01		

*Note:* Mann–Whitney *U*‐test was used for non‐normally distributed variables (all *p* > 0.05).

**Table 3:** Comparison of intervention characteristics between marital and control groups.

Variable	Group	Mean ± SD	*t* value	*p* value
Duration of marriage (years)	Intervention	20.28 ± 5.77	−0.256	*p* = 0.80
	Control	20.58 ± 5.95		
Age at marriage (years)	Intervention	21.84 ± 4.65	−0.519	*p* = 0.60
	Control	22.34 ± 4.74		

*Note:* Independent *t*‐test was used for normally distributed variables (all *p* > 0.05).

**Table 4:** Comparison of categorical demographic and clinical variables between intervention and control groups.

Variable	Category	Intervention (*n* = 50) *n* (%)	Control (*n* = 50) *n* (%)	Test statistic	*p* value
Number of pregnancies	1	4 (8)	5 (10)	*χ* ^2^ = 1.56	*p* = 0.82
	2	22 (42)	21 (42)		
	3	14 (28)	17 (34)		
	≥ 4	10 (20)	7 (14)		
Number of children	1	4 (8)	5 (10)	*χ* ^2^ = 1.99	*p* = 0.74
	2	22 (44)	21 (42)		
	3	15 (30)	18 (36)		
	≥ 4	9 (18)	7 (14)		
Number of births	1	4 (8)	5 (10)	*χ* ^2^ = 2.59	*p* = 0.63
	2	20 (42)	21 (42)		
	3	16 (28)	18 (36)		
	≥ 4	10 (14)	6 (12)		
Woman's education	Illiterate	7 (14)	5 (10)	*χ* ^2^ = 0.54	*p* = 0.97
	< Diploma	24 (48)	25 (50)		
	Diploma	17 (34)	18 (36)		
	Academic	2 (4)	2 (4)		
Spouse's education	Illiterate	2 (4)	0 (0)	*χ* ^2^ = 1.94	*p* = 0.67
	< Diploma	17 (34)	16 (32)		
	Diploma	23 (46)	24 (48)		
	Academic	8 (16)	10 (20)		
Economic status	Income greater than expenses	1 (2)	3 (6)	*χ* ^2^ = 5.19	*p* = 0.08
	Income less than expenses	13 (26)	22 (44)		
	Income equal to expenses	36 (72)	25 (50)		
Woman's employment	Housewife	25 (50)	23 (46)	*χ* ^2^ = 0.69	*p* = 0.16
	Employed	25 (50)	27 (54)		
Spouse's employment	Unemployed	4 (8)	0 (0)	Fisher's exact	*p* = 0.12
	Worker	17 (34)	19 (38)		
	Employee	7 (14)	12 (24)		
	Self‐employed	20 (40)	19 (38)		
	Retired	2 (4)	0 (0)		
Type of surgery	Partial hysterectomy	29 (58)	21 (42)	*χ* ^2^ = 2.56	*p* = 0.11
	Total hysterectomy	21 (42)	29 (58)		
Reason for hysterectomy	Cesarean‐related complications	2 (4)	3 (6)	Fisher's exact	*p* = 0.11
	Fibroid	24 (48)	22 (44)		
	Endometriosis	6 (12)	4 (8)		
	Hyperplasia	3 (6)	4 (8)		
	Irregular bleeding	15 (30)	17 (34)		

*Note:* Data are presented as *n* (%). Pearson's *χ* ^2^ test was used for most categorical variables; Fisher's exact test was applied when expected cell counts were < 5.

No significant between‐group differences were observed in mean age (*M* = 42.44 ± 4.24 vs. 42.98 ± 4.26; *p* = 0.47), age at disease onset (39.88 ± 5.06 vs. 41.36 ± 3.34; *p* = 0.21), or duration of illness (2.76 ± 1.92 vs. 2.44 ± 1.01; *p* = 0.57). Likewise, age at marriage (21.84 ± 4.65 vs. 22.34 ± 4.74; *p* = 0.60) and duration of marriage (20.28 ± 5.77 vs. 20.58 ± 5.95; *p *= 0.80) did not differ significantly between groups. Frequency distributions of education, economic status, employment, and surgical characteristics were also comparable (all *p* > 0.05).

### 3.2 Effect of the Intervention on Sexual Self‐Concept

At baseline, there were no significant differences between the two groups in the overall sexual self‐concept score (43.14 ± 3.58 vs. 42.50 ± 2.45; *p* = 0.20) or in any subscales, including sexual self‐efficacy, sexual anxiety, sexual satisfaction, and sexual desire (all *p *> 0.05).

At post‐intervention assessment, significant between‐group differences were observed across all dimensions of sexual self‐concept, favoring the intervention group. The total sexual self‐concept score increased from 43.14 ± 3.58 to 51.48 ± 2.61, compared with 44.18 ± 2.37 in the control group (*Z* = −8.28, *p* < 0.001) (Tables 5, 6). Specifically, sexual self‐efficacy (13.54 ± 1.63 vs. 11.14 ± 1.21), sexual satisfaction (14.92 ± 1.39 vs. 10.44 ± 1.31), sexual desire (13.64 ± 1.56 vs. 10.80 ± 1.22), and lower sexual anxiety (9.38 ± 1.62 vs. 11.80 ± 1.60) all showed significant differences favoring the intervention group (all *p* < 0.001) (Tables 7, 8).

**Table 5:** Comparison of total sexual self‐concept scores at baseline between intervention and control groups.

Variable	Group	Mean ± SD	*Z* value	*p* value
Total sexual self‐concept score	Intervention	43.14 ± 3.58	−1.28	*p* = 0.20
	Control	42.50 ± 2.45		

*Note:* Mann–Whitney *U*‐test was used for between‐group comparison at baseline.

**Table 6:** Comparison of total sexual self‐concept scores at post‐intervention between intervention and control groups.

Variable	Group	Mean ± SD	*Z* value	*p* value
Total sexual self‐concept score	Intervention	51.48 ± 2.61	−8.281	*p* < 0.001
	Control	44.18 ± 2.37		

*Note:* Mann–Whitney *U*‐test was used for between‐group comparison at post‐intervention.

**Table 7:** Comparison of total sexual self‐concept and its subscales at baseline between intervention and control groups.

Variable	Intervention (*n* = 50) Mean ± SD	Control (*n* = 50) Mean ± SD	*Z* value	*p* value
Sexual self‐efficacy	9.96 ± 1.19	10.02 ± 1.07	−0.07	*p* = 0.94
Sexual anxiety	13.28 ± 1.72	13.46 ± 1.54	−0.64	*p* = 0.52
Sexual satisfaction	9.70 ± 1.46	9.26 ± 1.41	−1.49	*p* = 0.14
Sexual desire	10.20 ± 1.79	9.76 ± 1.22	−1.37	*p* = 0.17
Total sexual self‐concept	43.14 ± 3.58	42.50 ± 2.45	−1.28	*p* = 0.20

*Note:* Mann–Whitney *U*‐test was used for between‐group comparisons at baseline (all *p* > 0.05).

**Table 8:** Comparison of total sexual self‐concept and its subscales at post‐intervention between intervention and control groups.

Variable	Intervention (*n* = 50) Mean ± SD	Control (*n* = 50) Mean ± SD	*Z* value	*p* value
Sexual self‐efficacy	13.54 ± 1.63	11.14 ± 1.21	−6.73	*p* < 0.001
Sexual anxiety	9.38 ± 1.62	11.80 ± 1.60	−6.13	*p* < 0.001
Sexual satisfaction	14.92 ± 1.39	10.44 ± 1.31	−8.58	*p* < 0.001
Sexual desire	13.64 ± 1.56	10.80 ± 1.22	−7.15	*p* < 0.001
Total sexual self‐concept	51.48 ± 2.61	44.18 ± 2.37	−8.28	*p* < 0.001

*Note:* Mann–Whitney *U*‐test was used for between‐group comparisons at post‐intervention.

ANCOVA indicated a significant post‐intervention effect of counseling on overall sexual self‐concept after controlling for baseline values, *F*_(1, 97)_ = 45.82, *p* < 0.001, η² = 0.32 (95% CI = [0.19, 0.46]. This indicates that approximately 32% of the variance in post‐intervention sexual self‐concept scores was attributable to the intervention after adjusting for baseline values.

### 3.3 Effect of the Intervention on Sexual Distress

Before the intervention, the two groups did not differ significantly in total sexual distress scores (43.14 ± 2.96 vs. 42.68 ± 2.72; *p *= 0.21) (Table 9). At post‐intervention assessment, sexual distress scores were lower in the intervention group (29.06 ± 2.64) than in the control group (42.04 ± 3.29) (*Z* = −12.98, *p* < 0.001) (Table 10). ANCOVA indicated a significant main effect of the intervention on sexual distress after adjusting for baseline levels, *F*_(1, 97)_ = 53.21, *p* < 0.001, η² = 0.35 (95% CI = (0.22, 0.48) (Table 11). This suggests that approximately 35% of the variance in post‐intervention sexual distress scores was explained by the intervention after controlling for baseline values.

**Table 9:** Comparison of sexual distress scores at baseline between intervention and control groups.

Variable	Intervention (*n* = 50) Mean ± SD	Control (*n* = 50) Mean ± SD	*Z* value	*p* value
Sexual distress	43.14 ± 2.96	42.68 ± 2.72	−1.25	*p* = 0.21

*Note:* Mann–Whitney *U*‐test was used for between‐group comparison at baseline.

**Table 10:** Comparison of sexual distress scores at post‐intervention between intervention and control groups.

Variable	Intervention (*n* = 50) Mean ± SD	Control (*n* = 50) Mean ± SD	*Z* value	*p* value
Sexual distress	29.06 ± 2.64	42.04 ± 3.29	−12.98	*p* < 0.001

*Note:* Mann–Whitney *U*‐test was used for between‐group comparison at post‐intervention.

**Table 11:** ANCOVA results for post‐intervention outcomes adjusting for baseline values.

Outcome	*F*_(1, 97)_	*p* value	Partial η²	95% CI for partial η²
Total sexual self‐concept	45.82	*p* < 0.001	0.32	[0.19, 0.46]
Sexual distress	53.21	*p* < 0.001	0.35	[0.22, 0.48]

*Note:* ANCOVA was conducted to assess the effect of the intervention on post‐intervention outcomes while controlling for baseline scores. Partial η² represents effect size.

### 3.4 Summary of Findings

Overall, psychosexual counseling based on the Schover bio‐psycho‐social model was associated with higher sexual self‐concept scores and lower sexual distress scores compared with routine postoperative care. These effects remained statistically significant after adjusting for baseline measures, with large effect sizes (η² > 0.30).

## 4 Discussion

The current study examined the impact of psychosexual counseling on sexual self‐concept and sexual distress among women who underwent hysterectomy and were referred to Kowsar Educational and Therapeutic Center in Urmia, Iran, during 2022–2023. Statistical analyses indicated that there were no significant demographic differences between the intervention and control groups, suggesting baseline similarity. The intervention significantly improved sexual self‐concept and reduced sexual distress, supporting the main hypothesis that psychosexual counseling can enhance sexual self‐concept and reduce sexual distress after hysterectomy.

The results reinforce the biopsychosocial perspective, which emphasizes that sexual health and recovery are determined by the interplay of biological, psychological, and social factors. From a biological standpoint, hysterectomy alters hormonal balance and pelvic anatomy, potentially affecting libido and sexual function. Psychosexual counseling helped participants understand these physiological changes, manage bodily responses, and improve sexual confidence. Psychologically, the intervention targeted negative cognitions and maladaptive beliefs surrounding femininity and body image. Participants showed improvements in sexual self‐efficacy and reductions in sexual anxiety, echoing previous findings by Mohamadnezhad et al. [34] and Rehan et al. [35], who demonstrated that cognitive‐behavioral and psychosexual interventions enhance self‐esteem and reduce distress in women who underwent hysterectomy. Socially, the counseling sessions encouraged open communication between partners and provided peer support, vital for rebuilding intimacy and relational stability.

These results are consistent with prior studies demonstrating that targeted psychosexual interventions improve sexual function and related sexual outcomes. Rehan et al. [35] and Shokre et al. [36] found that psychosexual or supportive counseling programs improved sexual quality of life and reduced depression in post‐hysterectomy women. Similarly, Elmoneim et al. [37] reported that group‐based interventions incorporating education, relaxation, and behavioral exercises enhanced sexual satisfaction and function, consistent with Roy's adaptive model, which conceptualizes adaptation as a multidimensional process. In the Iranian context, Goudarzi et al. [10] highlighted disruptions or inconsistencies in self‐concept cognition among women who underwent hysterectomy, recommending structured pre‐ and post‐surgical counseling—recommendations that are supported by the findings of the present study. Dangesaraki et al. [38] demonstrated that EX‐PLISSIT‐based sexual counseling improved desire and quality of life, paralleling our results, which indicated reductions in sexual anxiety and distress following structured counseling.

Conversely, Chow et al. [39] using the SEXHAB model among cancer patients, reported limited quantitative improvements but positive qualitative feedback, likely reflecting population and context differences.

Other complementary findings support the present conclusions. Gahlawat [40] identified hysterectomy as a psychologically traumatic experience altering identity and body image, while Hosseini et al. [41] showed that PRECEDE‐model education improved sexual function through behavior modification. EidFarrag et al. [42] and Abdelbaseer et al. [43] found that psychoeducational programs strengthened self‐esteem and marital communication. Collectively, these findings suggest that psychosexual counseling grounded in the biopsychosocial framework may offer a structured approach to supporting emotional and sexual adaptation following hysterectomy.

The clinical implications of this research are substantial. Psychosexual counseling may be considered as a component of post‐hysterectomy care plans. Healthcare systems and policymakers may benefit from acknowledging that women's post‐surgical adaptation extends beyond physical rehabilitation and requires attention to psychosocial and sexual dimensions. Providing structured counseling in hospitals and community clinics may contribute to improvements in sexual self‐concept and reductions in sexual distress. Culturally adapted, group‐based counseling models may be particularly suitable in societies where discussing sexual issues is stigmatized, as they can help normalize dialogue and reduce perceived stigma. This study's strengths include its randomized controlled design, the use of validated instruments (MSSQ‐SF and FSDS‐R), and a standardized intervention protocol delivered by trained professionals. Baseline similarity between groups further supported internal validity. However, the study had limitations: the short 1‐month follow‐up restricts conclusions about long‐term outcomes; reliance on self‐reported questionnaires may introduce response bias; and single‐center sampling may limit generalizability. Future studies may include longer follow‐ups, couple‐based interventions, and assessments of potential neuroendocrine correlates of post‐hysterectomy sexual recovery. Cross‐cultural comparisons may also help clarify the influence of sociocultural factors on psychosexual adaptation.

## 5 Conclusion

In conclusion, psychosexual counseling based on the biopsychosocial model was associated with significant improvements in self‐concept and reductions in sexual distress among women following hysterectomy. These findings highlight the relevance of addressing sexual and psychosocial dimensions during post‐hysterectomy recovery. Integrating such counseling into postoperative care may help bridge the gap between medical recovery and sexual well‐being, contributing to a more comprehensive approach to women's health care.

## Author Contributions

Z.M. drafted the manuscript. S.R. edited the manuscript. S.B. conceptualized and supervised the study and critically revised the manuscript. All authors read and approved the final version of the manuscript. Samira Barjasteh had full access to all of the data in this study and takes complete responsibility for the integrity of the data and the accuracy of the data analysis.

## Ethics Statement

This study was approved by the Ethics Committee of Urmia University of Medical Sciences (IR.UMSU.REC.1402.025; April 19, 2023) and registered in the Iranian Registry of Clinical Trials (IRCT20230507058109N1; July 15, 2023). All participants provided written informed consent in Persian.

## Consent

The authors have nothing to report.

## Conflicts of Interest

The authors declare no conflicts of interest.

## Transparency Statement

The lead author, Samira Barjasteh, affirms that this manuscript is an honest, accurate, and transparent account of the study being reported; that no important aspects of the study have been omitted; and that any discrepancies from the study as planned (and, if relevant, registered) have been explained.

## Data Availability

The data that support the findings of this study are available from the corresponding author upon reasonable request.
